# Effect of bisphenol A on human neutrophils immunophenotype

**DOI:** 10.1038/s41598-020-59753-2

**Published:** 2020-02-20

**Authors:** Wioletta Ratajczak-Wrona, Małgorzata Rusak, Karolina Nowak, Milena Dabrowska, Piotr Radziwon, Ewa Jablonska

**Affiliations:** 1 Department of Immunology, Medical University of Bialystok, ul. Waszyngtona 15A, 15-269 Bialystok, Poland; 2 Department of Hematological Diagnostics, Medical University of Bialystok, ul. Waszyngtona 15A, 15-269 Bialystok, Poland; 3 Regional Centre for Transfusion Medicine, Bialystok, ul. M. Skłodowskiej - Curie 23, 15-950 Bialystok, Poland

**Keywords:** Immunology, Environmental sciences

## Abstract

Neutrophils (PMN) play a key role in eliciting congenital immune response. These cells are equipped with specific receptors that are located on the surface of their cell membrane. These receptors produce various signals which in turn help in the effective functioning of PMN. The activity of these cells may be modified by factors of endo- and exogenous origin, including xenoestrogens such as bisphenol A (BPA). The aim of this study was to evaluate the effect of BPA on the expression of CD11c, CD14, CD15, CD16, CD62L and CD284 compounds on the surface of neutrophils in women and men. The study material included PMN isolated from the whole blood. The cells were incubated in the presence of BPA and/or LPS. Flow cytometry technique was used to evaluate the expression of CD antigens. Studies of these receptors indicate that BPA, at a concentration corresponding to the serum level of this compound in healthy subjects as well as at higher doses, induces changes in the immunophenotype of PMN, which may lead to immunity disorders associated with the dysfunction of these cells. Moreover, the observed effects of xenoestrogen on the expression of CD11c, CD14, CD15, CD16, CD62L and CD284 differentiation markers on these cells are sex-independent.

## Introduction

There has been an increase in the number of allergic diseases, diabetics, obese individuals, and cases of endocrine, neurological and sexual disorders worldwide since the twentieth century and continues to show an increasing trend. This observation can be attributed to the rapidly growing chemical pollution of the environment. Humans are constantly exposed to natural and synthetic chemical compounds. Among them, xenoestrogens, estrogen-like compounds of exogenous origin endocrine disrupting compounds (EDCs), play an important role. They have the ability to interact with the hormonal system and modulate its functions in a manner that is characteristic for estrogens. Sources of xenoestrogens include some pharmaceutical compounds, metals, detergents, and chemicals used to harden plastics, such as bisphenol A (BPA)^1–7^. The popularity of plastic products has lead to the widespread use of BPA. BPA easily penetrates into food and beverages at elevated temperatures or as a result of damage caused at the time of packaging. Humans are exposed to this compound mainly through food products, and the absorption is found to be particularly high in children. BPA is known to exert harmful effects on human health even at low concentrations. This factor disturbs the hormonal homeostasis of the organism, leading to infertility and cancers^4,5,8–10^.

Bisphenol A can affect the body functions through complex and still not fully understood mechanisms, including both the interaction with receptors and the influence on the permeability of cell membranes. There is evidence to prove that low levels of BPA cause negative effects on the female hormone estrogen, consequently causing a disruption of the hormonal balance in the body. The chemical structure of BPA is similar to that of phenol, thus enabling it to act as an agonist or antagonist. Its action is probably dependent on the content of estrogens^1,3,8^. At lower concentration of estrogens in the body, it exhibits agonist characteristics, while at higher concentrations, it behaves as an antagonist^1,11^. BPA can bind to estrogen receptors, estrogen-related receptors, aryl hydrocarbon receptors, and peroxisome proliferator*-*activated receptors. Owing to its ability to interact with many types of receptors, BPA has a wide impact on immune system regulation^12^.

Despite the conductance of numerous scientific studies on BPA, its effects on PMN are still unclear^13,14^. PMN, the largest pool of peripheral blood leukocytes, are the first line of defense against pathogens such as bacteria, fungi, and some viruses. They also play a crucial role in providing protection against cancer. These cells contain specific receptors on the surface of their cell membrane, which enable them to interact with the extracellular environment and with other cells. These include, inter alia, receptors for Fc fragment of immunoglobulins (CD16), complement components (Complement receptor *-* CR), adhesion molecules (CD11c, CD15, CD62L), LPS (CD14), as well as receptors for hormones including estradiol (NR3). The receptors respond to the external stimuli by providing appropriate signals that further help in the activation and functioning of various intracellular transmission pathways, which include pathogen recognition, opsonization, phagocytosis, complement activation, and initiation of PMN death through apoptosis^15–18^.

Our previous studies have shown the influence of BPA on the mechanism of intracellular oxygen-dependent (related to nitric oxide) and oxygen-independent (related to serine protease release) killing of PMN in both women and men^19,20^. Moreover, the observed effects of xenoestrogen activity in these cells were dependent on the sex. Therefore, we undertook further studies in women and men to determine the influence of BPA on the expression of CD11c, CD14, CD15, CD16, and CD62L antigens on the surface of PMN, which are known to participate in the basic functions related to the diagnosis and elimination of pathogens.

Due to the fact that the mechanisms involved in EDC action are very complex and conditioned by the ability to act simultaneously on many receptors, which results in observed differential effects, it is difficult to unequivocally determine the toxicity of these compounds. EDCs show a non-monotonic dose–effect relationship, in which both very low and high doses can provide maximum response, while no effect is observed at intermediate doses. This relationship is often presented as a U-shaped curve. In the case of EDCs, a dose–effect relationship may also be presented as an inverted U-shaped curve, where intermediate doses confer the maximum effect^21–23^. Based on the literature data^24–26^, our use of high BPA concentrations (1.5–12 μM) is intended to implement modern standards for immunotoxicological studies which would further allow to determine the linear (or nonlinear) dose–effect relationship in terms of BPA effect on the expression of the molecules studied. In previous studies undertaken in our laboratory, BPA was present in 97% of the analyzed serum samples of healthy subjects, and its mean concentration was found to be 14.94 nM in women and 17.17 nM in men^19^.

## Material and Methods

### Isolation and incubation of PMNs

The tested blood was obtained from 15 volunteer donors from the Regional Centre for Transfusion Medicine (Bialystok, Poland). Famales were in follicular phase of menstrual cycle. All blood donors were in age 20–25 years, do not smoke, do not alcohol consume 48 hours prior blood donation. They do not have any chronic diseases and immunological deficiencies in medical history^19,20^.

All donors have been informed regarding the study objectives and methodology, and have provided written consent for participation in the study.

The approval for the conductance of the study titled “Assessment of bisphenol A influence on the immunophenotype of human peripheral blood leukocytes” (Resolution no.: R-I-002/95/2018) was granted by the Bioethics Committee of the Medical University of Białystok. All of the experiments were performed in accordance with good laboratory practice.

**Figure 1:**
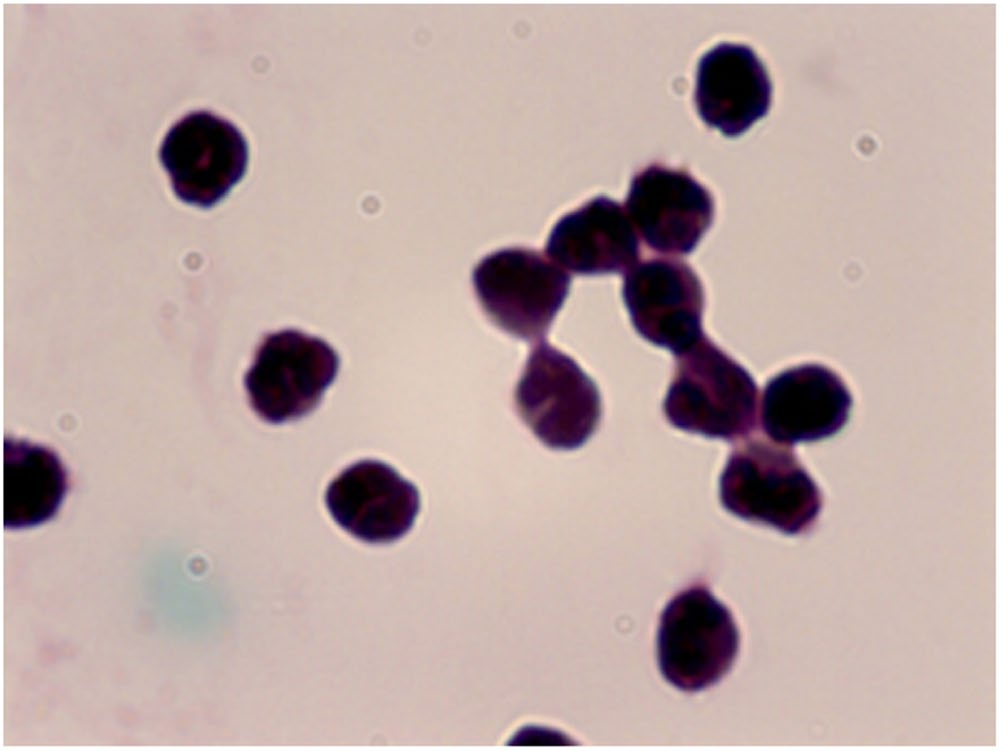
The 97% pure neutrophils after isolation in density centrifugation and positive selection with CD16 beads. Neutrophils were stained by May-Grünwald-Giemsa and assessed in the light microscope. Malignation × 100.

The study material included venous blood collected with an anticoagulant (heparin, Polfa, Lodz, Poland) and without an anticoagulant for serum purpose. From each donor, 4 ml of venous blood was drawn and overlaid on Polymorphprep™ (AXIS-SHIELD PoC AS, Oslo, Norway). Neutrophils were isolated by centrifuging the sample at 400 × *g* for 30 min in a density gradient. Cells were counted in a Bürker chamber after staining the cell nuclei with Türk’s solution. Subsequently, purity of the cell suspension was assessed by performing “thick drop” method by utilizing May-Grünwald and Giemsa stains. The samples of donor cells that demonstrated neutrophil purity of more than 85% were subjected to a subsequent stage of cell isolation. During positive separation process, we were able to effectively isolate neutrophils in accordance with the protocol recommended by Miltenyi Biotec company using MACS® Separator and CD16 MicroBeads (catalog no. 130-045-701) (Fig. 1).

**Figure 2:**
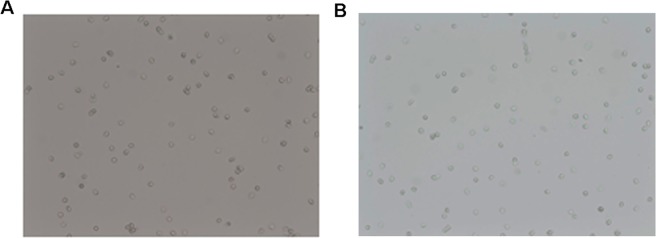
The 98% survival of the neutrophils stained with trypan blue. Cells were counted in the light microscope. Malignation × 100. (**A**) cells after isolation; (**B**) cells affter 20 hours incubation.

The viability of neutrophils was determined using trypan blue staining (Lachema), which does not penetrate live cells but dead cells (late apoptotic and necrotic) are permeable to the stain and appear blue. Neutrophils stained with trypan blue were counted manually under a light microscope. Neutrophil viability was analyzed in the preparations developed directly after isolation, as well as in those obtained after 20 hours of incubation (Fig. 2).

The isolated neutrophils were cultured on the HBSS medium (Invitrogen, Carlsbad, CA), which was enriched with donor’s serum and antibiotics (penicillin and streptomycin (Polfa Trachomin SA, Warsaw, Poland)). The cells were incubated for 20 hours in sterile plates at 37 °C in 5% CO_2_ (Nuaire^TM^ US Autoflow, Plymouth, MN). Neutrophils can survive in the peripheral blood for approx. 8 hours, and then move to the tissues where they can sustain for 2 to 3 days. Recent studies have demonstrated that their survival period may be much longer—even up to about 90 hours^27^. Based on these data, as well as considering the outcomes of earlier research on bisphenol A, we undertook the following study, wherein we exposed neutrophils to bisphenol A (at concentrations 12 µM, 6 µM, 3 µM, 1.5 µM, and 16 nM) or LPS (at concentration 10 µg/ml) for 20 hours^19,20^.

Bisphenol A (catalog no. 42088-100 MG, Sigma-Aldrich) employed in this study is 99% pure and is provided with Certificate of Analysis: Certified Reference Material. Lipopolysaccharides (LPS) used for the study (catalog no. L3129, Sigma-Aldrich) were obtained from Escherichia coli O127: B8 and purified by phenol extraction to 97% purity. The LPS fraction thus extracted contains <3% of other proteins, this compound has also been provided with the Certificate of Analysis.

### Flow cytometry analysis

To 50 µl of the cells suspended in PBS, 20 µl of each of the following monoclonal antibodies was added: anti-CD11c, anti-CD14, anti-CD15, anti-CD16, anti-CD62L, and anti-CD284 (TLR4). After 30 minutes of incubation in the dark, the samples were rinsed with PBS by centrifuging for 5 minutes. The results were analyzed for 30 minutes on a flow cytometer (Canto II, Becton Dickinson) using FACSDiva software.

### Statistics

Statistical analysis was done using Statsoft Statistica version 13.3. Data was presented in terms of mean ± S.E or mean ± SD. The normal distribution of data was tested by the Kolmogorov-Smirnov test. The CD data were compared with the Mann-Whitney U test. Differences were considered statistically significant when the *P* < 0.05.

## Results

### Evaluation of CD11c, CD14, CD15, CD16, and CD62L expression in female and male neutrophils

**Figure 3:**
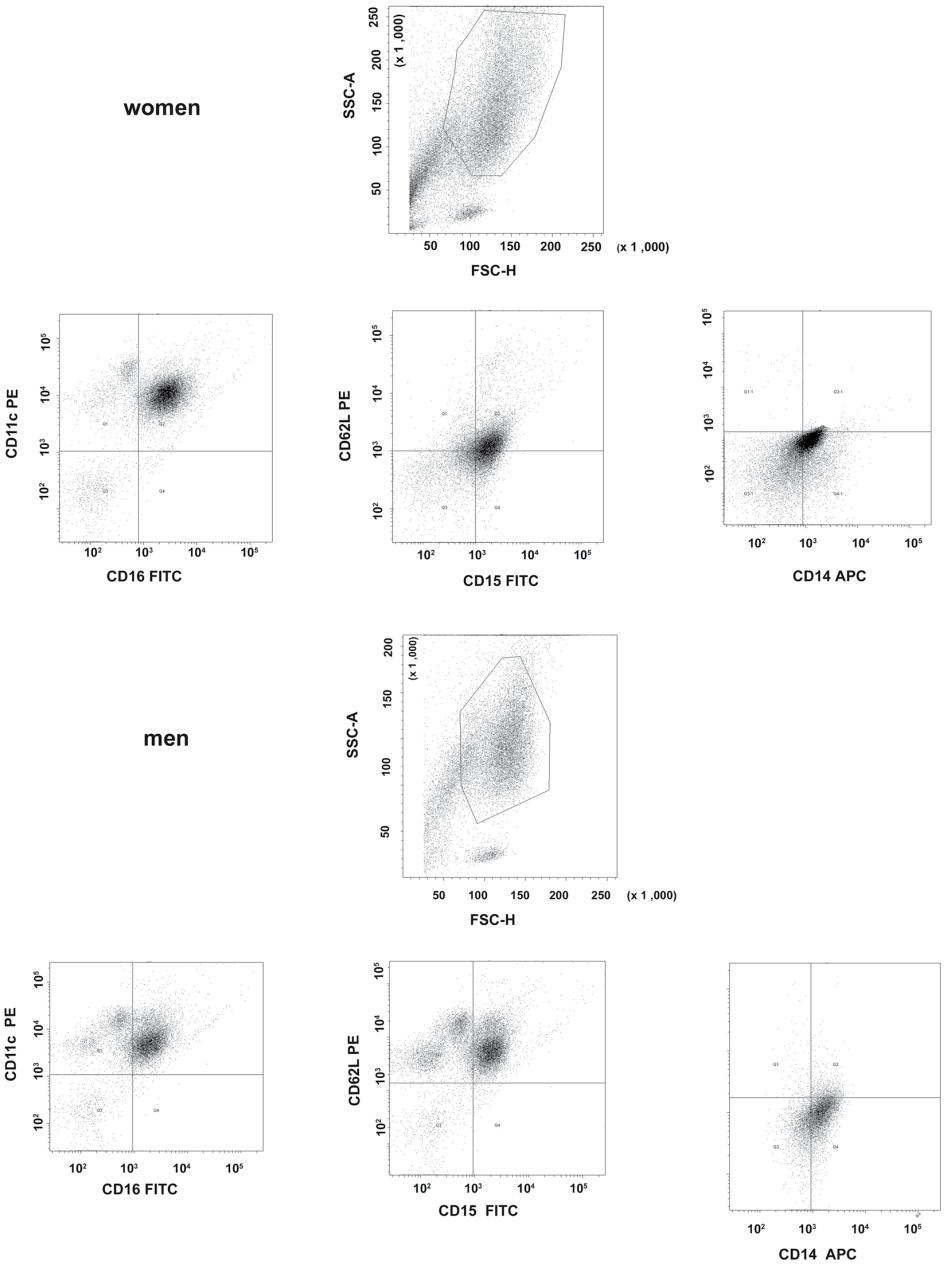
Representative FCAS plots demonstrating of CD antigens expression on the PMN.

The expression of CD11c, CD14, CD15, CD16, and CD62L cell surface antigens was demonstrated on the cell membrane of PMN in women and men (Fig. 3).

**Figure 4:**
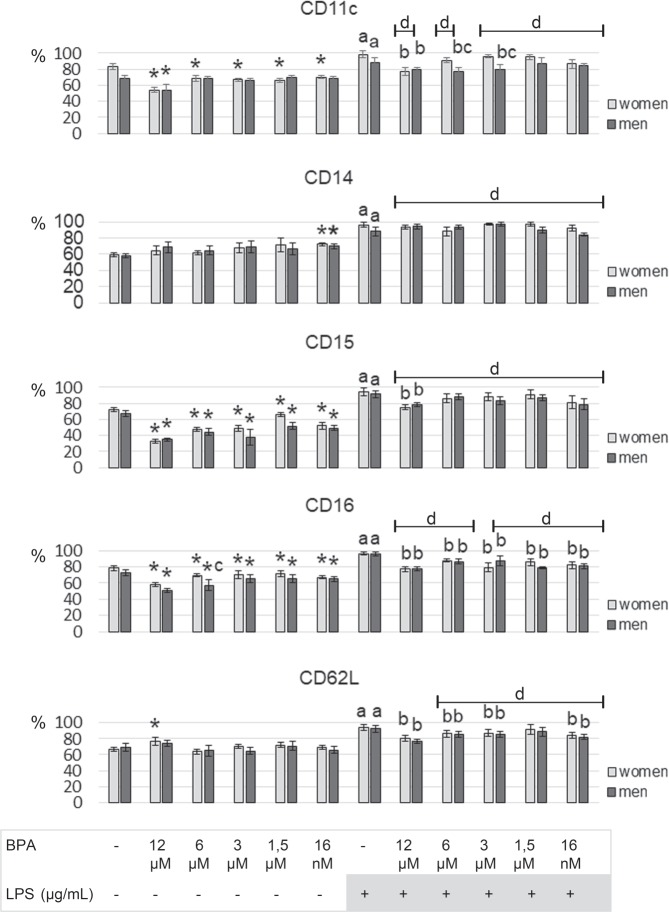
Alterations in CD11c, CD14, CD15, CD16 and CD62L in human PMN. PMN were treated for 20 hours with BPA (12 μM, 6 μM, 3 μM, 1.5 μM or 16 nM) and/or LPS (10 µg/ml). % **-** percentage share of cells with positive antigen expression; value significantly different between * – cells without and with BPA (*p* < 0.05); (**a**) cells incubated only with LPS and cells incubated without BPA and LPS (*p* < 0.05); (**b**) cells incubated only with LPS and cells simultaneously incubated with LPS and BPA (*p* < 0.05); (**d**) cells incubated only with BPA and cells incubated with LPS and BPA (*p* < 0.05); (**c**) cells collected from women and men (*p* < 0.05).

Exposure of female PMN to BPA (at concentrations of 12 μM, 6 μM, 3 μM, 1.5 μM, or 16 nM) led to a decrease in the percentage of PMN expressing CD11c, CD15, and CD16 markers compared to cells not treated with xenoestrogen. On the other hand, the highest BPA concentration resulted in a higher percentage of PMN showing CD62L expression compared to non-xenoestrogen-treated cells. However, in the presence of other applied BPA concentrations (6 μM, 3 μM, 1.5 μM, or 16 nM), no changes in the percentage of PMN expressing CD62L antigen were found (Fig. 4).

Exposing the PMN of female as well as male participants to 16 nM BPA led to an increase in the percentage of CD14-expressed PMN compared to non-xenoestrogen-treated cells. In the presence of other applied BPA concentrations, no changes in the percentage of PMN with CD14 expression at the surface were found (Fig. 4).

Similar to the results obtained for female PMN, incubation of male cells with BPA (at all applied concentrations) showed a lower percentage of PMN expressing CD15 and CD16 markers on their surface compared to PMN incubated without xenoestrogen. The highest BPA concentration (12 μM) caused a decrease in the percentage of PMN with CD11c antigen. The exposure of PMN to other BPA concentrations (6 μM, 3 μM, 1.5 μM, or 16 nM) showed no changes in the percentage of PMN with CD11c antigen. No changes in the percentage of PMN with CD62L expression were observed in the presence of all BPA concentrations (Fig. 4).

Stimulation of PMN with LPS in women and men caused an increase in the percentage of PMN expressing CD11c, CD14, CD15, CD16, and CD62L antigens compared to the cells not stimulated by LPS and not exposed to BPA (Fig. 4).

A lower percentage of PMN with CD11c and CD15 expression was found in female PMN following exposure to LPS and 12 μM concentration of BPA compared to the cells stimulated by LPS. However, no changes were observed in the percentage of PMN expressing these markers in the presence of LPS and other applied BPA concentrations (6 μM, 3 μM, 1.5 μM, or 16 nM) (Fig. 4).

In contrast to the results obtained in females for PMN, incubation of male cells with BPA at 12, 6, or 3 μM concentrations led to a decrease in the percentage of PMN with CD11c expression compared to LPS-stimulated cells. Moreover, similarly as in women, a reduced percentage of PMN with CD15 expression was observed in the presence of LPS and the highest BPA concentration used (Fig. 4).

When female and male PMN were exposed simultaneously to both LPS and xenoestrogen BPA (12 μM, 6 μM, 3 μM, 1.5 μM, or 16 nM) stimulators, no changes in the percentage of PMN with CD14 expression were found compared to cells stimulated by LPS only (Fig. 4).

Furthermore, simultaneous stimulation of PMN of both women and men with LPS and BPA at 12, 6, and 3 μM concentrations led to a decrease in the percentage of PMN expressing CD16 and CD62L antigens compared to LPS-stimulated cells only. Moreover, when LPS and BPA were used at 1.5 μM concentration, a decrease in the percentage of CD16+ cells was observed in both sexes (Fig. 4).

In female and male neutrophils, a reduction in the percentage of neutrophils with CD16 and CD62L expression was observed after treatment with LPS and BPA (16 nM) as compared to the cells only stimulated with LPS (Fig. 4).

Simultaneous use of LPS and BPA (at all concentrations) led to a higher percentage of PMN with CD14 and CD15 expression in women and men compared to the cells exposed to BPA only. In addition, an increase in the percentage of PMN with CD62L expression was also observed in the cells of both sexes exposed to LPS and BPA (6 μM, 3 μM, 1.5 μM, or 16 nM) (Fig. 4).

Stimulation of the cells of both sexes with LPS and BPA (at all concentrations) led to an increase in the proportion of PMN with CD11c antigen expression, except for male cells exposed to LPS and 6 μM concentration of BPA, compared to cells treated with BPA only (Fig. 4).

In addition, incubation of PMN of both sexes with LPS and BPA (at all concentrations) led to an increase in the proportion of PMN with CD16 antigen expression, except for female cells exposed to LPS and 3 μM BPA, compared to the cells treated with BPA only (Fig. 4).

The analysis of the results, based on the sex of the patient, did not show any difference in the expression of CD11c, CD14, CD15, CD16, and CD62L antigens on the surface of PMN (not stimulated with LPS and not exposed to BPA, exposed only to BPA, or stimulated only with LPS) between women and men. On the other hand, a lower percentage of PMN with CD11c expression in the presence of LPS and BPA (at the concentrations of 6 and 3 μM) was found in men compared to women. No changes were observed in the percentage of PMN expressing CD14, CD15, CD16, and CD62L antigens between women and men (Fig. 4).

**Figure 5:**
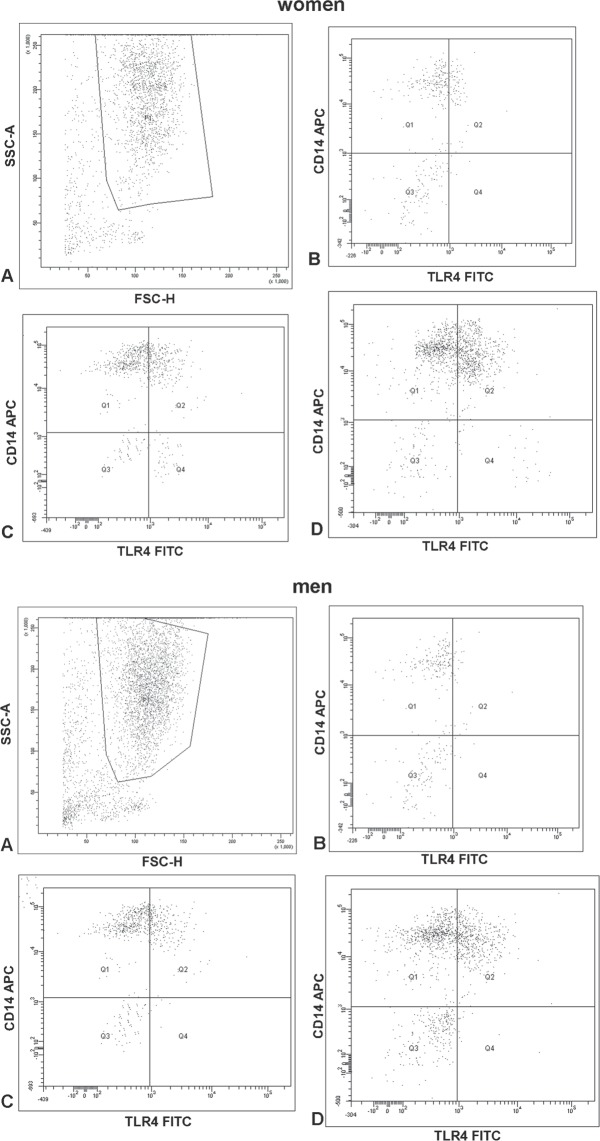
Representative FCAS plots demonstrating of CD antigens (CD14 and CD284 (TLR4)) expression on the PMN: (**A**) PMN; (**B**) PMN without BPA and LPS; (**C**) cells incubated only with BPA (16 nM); (**D**) cells incubated only with LPS.

Considering the effect of the lowest concentration of bisphenol A on CD14 expression of neutrophils, we also conducted a study to assess its impact on TLR4 expression. Incubation of neutrophils of both sexes with BPA (16 nM) resulted in an increased percentage of neutrophils with TLR4 receptor expression as compared to the cells not exposed to BPA (Fig. 5, Table 1). Stimulation with LPS of cells obtained from both sexes resulted in an elevated percentage of neutrophils with TLR4 receptor expression as compared to the non-stimulated cells with LPS (Fig. 5, Table 1). No changes were observed in the percentage of PMN expressing CD284 antigens between women and men.

**Table 1:** Alterations in TLR4 (CD284) in human PMN. PMN were treated for 20 hours with BPA (16 nM) or LPS (10 µg/ml).

	TLR4 (CD284)	
	women Mean ± SD	men Mean ± SD
PMN	6.8 ± 1.92	5.9 ± 1.97
PMN + BPA (16 nM)	16.75* ± 4.33	14.1* ± 4.21
PMN + LPS	31.1^a^ ± 7.23	26.1^a^ ± 7.52

Value significantly different between * – cells without and with BPA (*p* < 0.05); ^a^ – cells incubated only with LPS and cells incubated without BPA and LPS (*p* < 0.05).

## Discussion

Functional disorders of phagocytes often lead to recurrent infections, most frequent among them being purulent and fungal respiratory tract infections, as well as diseases related to subcutaneous tissue, skin, mucous membranes, and deep organ abscesses^28,29^.

The results of this study indicate that BPA acts by inducing changes in the expression of differentiation antigens like CD11c/CD18, CD14, CD15, CD16, and CD62 on the surface of PMN and subsequently changing the immunophenotype of human PMN in both women and men. Moreover, the observed changes in the PMN antigens were not found to be sex-specific.

The influence of BPA on the phenotype of nonspecific response cells, including monocytes, was also studied by Zbucka-Kretowska *et al*. They demonstrated that the culturing of human monocytes with BPA led to an increase in the percentage of classical subpopulations (CD14++ CD16−) of monocytes, without significantly affecting the percentage of nonclassical subpopulations (CD14++ CD16+ and CD14+ CD16++) of monocytes^30^.

Our own study demonstrated that BPA leads to a decrease in the percentage of PMN with CD11c expression in both women and men, which confirms the significant influence of this xenoestrogen on this glycoprotein. The demonstrated changes in the expression, and thus binding, of this receptor for fibrinogen and C4 complement component on the examined cells may lead to abnormal adhesion and phagocytosis of iC3b-coated molecules, as well as may induce phagocytosis without the participation of complement components in subjects exposed to this compound^31,32^.

In the present study, a trend similar to CD11c glycoprotein expression was observed for changes in the percentage of PMN with CD16 antigen expression. The decreased receptor expression for Fc Ig fragment on BPA-treated PMN may consequently lead to impaired phagocytosis induction as well as cell activation, inter alia, to antibody*-*dependent cellular cytotoxicity (ADCC). Moreover, alteration in the expression of CD16 on PMN may contribute to the dysfunction of these cells in the regulation of immune response, antigen presentation, as well in the secretion of inflammatory reaction mediators such as IL-1, IL-6, and TNF-α after binding to immune complexes^33–36^. The results of our study with regard to changes in the CD16 antigen expression are consistent with those reported by earlier studies. A decrease in CD16 expression on Natural killer (NK) cells due to the activity of endocrine-disrupting compounds as well as deterioration in the ability of PMN to phagocytose cells was observed under the influence of BPA^37–40^.

In addition, the study of PMN of both sexes exposed to different concentrations of BPA also showed a decrease in the percentage of PMN with CD15 antigen expression (stage-specific embryonic antigen 1), which may ultimately lead to abnormalities in various functions of these cells, including adhesion, phagocytosis, oxygen explosion, and degranulation^41^. Due to the wide involvement of CD15 molecule in the immune response, it seems particularly worrying that we observed changes in the expression of this molecule in the presence of the lowest xenoestrogen concentration, which corresponds to the average concentration of BPA determined in the human bloodstream (16 nM)^19^.

Moreover, overexpression of CD14, observed in this study, following exposure to the lowest concentration of bisphenol A (16 nM) may result in an elevated risk of systemic inflammation during infection with Gram-negative bacteria in individuals exposed to this xenoestrogen. There is evidence that the excessive expression of CD14 in transgenic mice increases their susceptibility to endotoxic shock and an increase in SCD14 in humans is related to increased mortality due to shock caused by G− and G+ bacteria^42^.

In the light of the available data, which shows that the key step in the production of inflammatory mediators is the activation of the “endotoxin receptor complex” (CD14 and MD2 molecules and Toll 4 receptor), the ability of BPA to activate the TRL4 receptor, as demonstrated by us as well as other authors, seems to be of particular importance^43^. The interactions of BPA with CD14 and TRL4 may lead to an increased expression of proinflammatory genes and thereby cytokine synthesis^44^. On the other hand, the interaction of BPA with CD14 may contribute to the disturbed internalization of TLR4 dependent on CD14 protein^45^.

In the presented study, observed increase in the percentage of PMN in women with CD62L (L-selectin) expression in the presence of the highest concentration of BPA, which may lead to increased interaction of these cells with the vascular endothelium, seems surprising^46,47^. Different results were presented by Tӧrӧk *et al*., who showed that the expression of this molecule on the PMN surface decreased significantly under the influence of even a slight stimulation, whereas no change in the expression was noted on the monocyte surface^48^.

Some studies have reported that PMN of people chronically exposed to endocrine-disrupting compounds, such as dichlorodiphenyltrichloroethane (DDT), show a decreased ability to chemotaxis, adhesion, phagocytosis, and oxygen-dependent killing. Moreover, it was observed that the dysfunction of these cells was associated with an increase in the incidence of infectious diseases, particularly upper respiratory tract infections, in this group of people^49^.

The results of our study showed that stimulation of PMN of both sexes with endotoxin (the main component of the outer membrane of Gram-negative bacteria) leads to an increased percentage of the cells expressing CD11c, CD14, CD15, CD16, and CD62L antigens, which suggests a proper development of inflammatory response to bacterial toxin. Similar observations were made by Rodeberg *et al*., who observed an increase in CD14 expression on PMN stimulated by LPS^50^. Different results were reported by Vega *et al*. with regard to CD62L marker^51^. They did not observe any changes in the expression of this selectin on PMN as a result of LPS stimulation. Kishimoto *et al*., however, demonstrated reduced CD62L expression on PMN following stimulation with the endotoxin^52^.

In order to evaluate the potential influence of BPA on the inflammatory process, PMN of women and men were concurrently stimulated with LPS and xenoestrogen. The observed decrease in the percentage of PMN expressing CD11c, CD16, and CD62L antigens in both sexes suggests that xenoestrogen may prevent rapid removal of the pathogen factor and consequently lead to the development of an inflammatory process. This etiology seems to be dangerous in pregnant women, who are particularly exposed to Gram-negative bacterial infections^53,54^. BPA alone can lead to a number of adverse effects in the offspring, including carbohydrate metabolism disorders^55^, fertility disorders^56^, and nervous system abnormalities^57^.

However, the study conducted in our laboratory did not show any influence of BPA on the percentage of PMN with CD14 expression after stimulation with LPS. This situation may be probably caused by higher affinity of bacterial endotoxin to TLR receptors compared to BPA^58,59^. LPS binds to a protein molecule (LPS binding protein) present in the serum, which in turn transports and transfers it to CD14. This receptor is anchored to the membrane by a glycosylphosphatidylinositol connector and is not capable of transmitting a signal by itself; only after the formation of a complex with TLR4, the cell can be activated^60,61^. On the other hand, the question arises as to whether we would also observe changes in the percentage of PMN with CD14 expression if these cells were exposed simultaneously to BPA and another component of the bacterial cell wall, which is lipoteichoic acid (LTA) from Gram-positive bacteria. There are data reporting that LTA induces the activation of PMN with CD14 expression, independent of binding with the Toll-like receptors, TLR2 or TLR4^62^.

In conclusion, it has already been established that BPA at a concentration corresponding to the serum level of this compound in healthy subjects as well as at higher doses affects the immunophenotype of PMN, which may subsequently lead to immunodeficiency disorders associated with dysfunction of these cells in subjects exposed to this compound. Moreover, the observed effects of xenoestrogen on the expression of CD11c, CD14, CD15, CD16, and CD62L differentiation markers on these cells are sex-independent.

The effects of BPA activity on the reproductive system and neonatal development have been widely recognized. However, there is little information with regard to its effects on the cells of the immune system, especially PMN. A thorough understanding of the mechanism of BPA action on PMN together with a description of the role of estrogenic receptors in these cells will allow for a realistic assessment of the risks resulting from wide exposure to this xenoestrogen. Therefore, further research is needed to determine the possible effects of BPA on the functioning of important immune cells, such as PMN.

### Ethical approval and consent to participate

The Ethics Committee of the Medical University of Bialystok (R-I-002/95/2018) approved this study. Informed consent was obtained from all participants prior to blood donations. All of the experiments were performed in accordance with good laboratory practice.

## Data Availability

The datasets used and/or analyzed during the current study are availablefrom the corresponding author on reasonable request.
